# P-1653. Current Prescribing Practices and Guideline Concordance for the Treatment of Community-Acquired Bacterial Pneumonia (CABP) across Outpatient and Urgent Care Visits

**DOI:** 10.1093/ofid/ofae631.1819

**Published:** 2025-01-29

**Authors:** Tomefa E Asempa, Tyler Ackley, Kristin E Linder, David P Nicolau

**Affiliations:** Hartford Hospital, Hartford, CT; Hartford Hospital, Hartford, CT; Hartford HealthCare, Hartford, Connecticut; Hartford Hospital, Hartford, CT

## Abstract

**Background:**

Resources and support for antibiotic stewardship programs (ASP) have focused on hospitalized patients and there is minimal prescribing data and interventions among outpatients. We aimed to assess the treatment of CABP in adults in order to develop interventions to improve antibiotic utilization.

**Figure:**
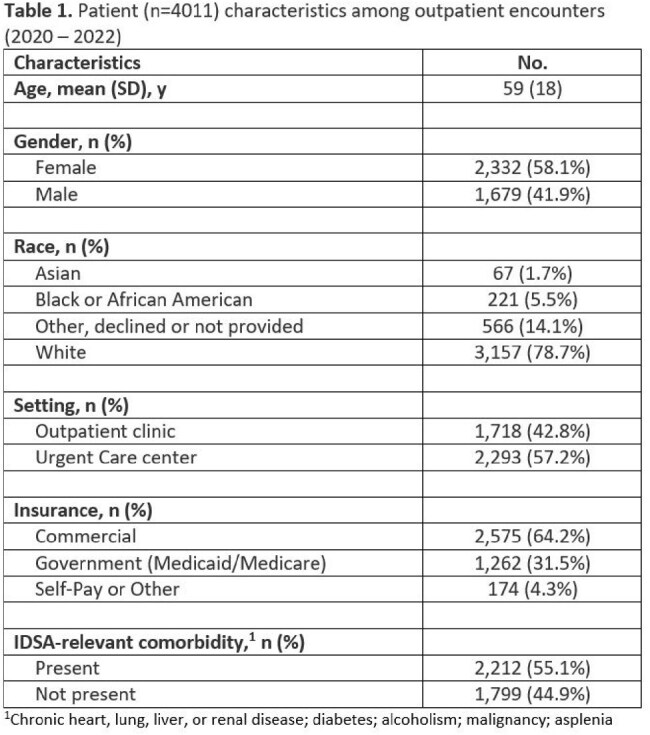

**Methods:**

This retrospective study (2022-2022) included patients within a large healthcare system presenting to outpatient clinics (n=120) and affiliated Urgent Care Centers (n=24) across Connecticut. Patient encounters were identified via CABP ICD-10 codes and IDSA relevant patient comorbidities (chronic heart, lung, liver, or renal disease; diabetes; alcoholism; malignancy; asplenia) were extracted from patient problem lists. Primary outcome was to describe the proportion of patients receiving concordant therapy (antibiotic choice and prescription of monotherapy or combination therapy depending on presence of comorbidity) per IDSA guideline and local antibiogram.

**Results:**

Patient characteristics are listed in Table 1. Overall guideline concordance rate was 36.6% (1,468/4,011 antibiotic orders). Among orders in patients with comorbidities (n=2,212), a large proportion received discordant therapy (n=1,417; 64.1%) via monotherapy particularly doxycycline (DOX) or azithromycin (AZM). Among appropriate combination therapy orders (n=370) in patients with comorbidities, the most common pairings were amoxicillin-clavulanate plus AZM (160/370; 43.2%) followed by amoxicillin-clavulanate plus DOX (86/370; 23.2%). 10.5% (232/2,212) received a guideline concordant respiratory fluoroquinolone monotherapy.

Among antibiotic orders in patients with no comorbidity (n=1,799), 1,126 (62.6%), orders were discordant largely due to inappropriate monotherapies (742/1,126; 65.9%) in particular AZM monotherapy (352/1,126; 31.3%) despite local pneumococcal resistance rates >25%.

**Conclusion:**

Guideline concordance was low, primarily due to lack of combination therapy in patients with comorbidities, as well as use of AZM monotherapy despite high resistance rates. Overall, these data will be instrumental in our current efforts to initiate an outpatient ASP and deploy interventions to improve care across the continuum of care.

**Disclosures:**

**Tomefa E. Asempa, PharmD**, FDA/CDER: Grant/Research Support|Paratek: Grant/Research Support|Shionogi: Grant/Research Support|Spero: Grant/Research Support|VenatoRx: Grant/Research Support **David P. Nicolau, PharmD**, CARB-X: Grant/Research Support|Innoviva: Grant/Research Support|Innoviva: Honoraria|Merck: Advisor/Consultant|Merck: Grant/Research Support|Merck: Honoraria|Pfizer: Advisor/Consultant|Pfizer: Grant/Research Support|Pfizer: Honoraria|Shionogi: Advisor/Consultant|Shionogi: Grant/Research Support|Shionogi: Honoraria|Venatorx: Grant/Research Support

